# Oligonucleotide Probes for ND-FISH Analysis to Identify Rye and Wheat Chromosomes

**DOI:** 10.1038/srep10552

**Published:** 2015-05-21

**Authors:** Shulan Fu, Lei Chen, Yangyang Wang, Meng Li, Zujun Yang, Ling Qiu, Benju Yan, Zhenglong Ren, Zongxiang Tang

**Affiliations:** 1 Province Key Laboratory of Plant Breeding and Genetics, Sichuan Agricultural University, Wenjiang, Chengdu 611130, Sichuan, People’s Republic of China; 2 Agronomy College, Sichuan Agricultural University, Wenjiang, Chengdu 611130, Sichuan, People’s Republic of China; 3 School of Life Science and Technology, University of Electronic Science and Technology of China, Chengdu 610054, Sichuan, People’s Republic of China

## Abstract

Genomic *in situ* hybridization (GISH) has been widely used to detect rye (*Secale cereale* L.) chromosomes in wheat (*Triticum aestivum* L.) introgression lines. The routine procedure of GISH using genomic DNA of rye as a probe is time-consuming and labor-intensive because of the preparation and labeling of genomic DNA of rye and denaturing of chromosomes and probes. In this study, new oligonucleotide probes Oligo-1162, Oligo-pSc200 and Oligo-pSc250 were developed. The three new probes can be used for non-denaturing fluorescence *in situ* hybridization (ND-FISH) assays and replace genomic DNA of rye as a probe to discriminate rye chromosomes in wheat backgrounds. In addition, previously developed oligonucleotide probes Oligo-pSc119.2-1, Oligo-pSc119.2-2, Oligo-pTa535-1, Oligo-pTa535-2, Oligo-pTa71-2, Oligo-pAWRC.1 and Oligo-CCS1 can also be used for ND-FISH of wheat and rye. These probes have provided an easier, faster and more cost-effective method for the FISH analysis of wheat and hybrids derived from wheat × rye.

Rye (*S. cereale* L.) has been used in wheat (*T. aestivum* L.) breeding programs as a source of genes for resistance to abiotic stress, diseases and insects^1^,^2^,^3^,^4^,^5^. Wheat-rye 1BL·1RS translocated chromosomes have been widely used to develop wheat cultivars^6^. In order to verify presence of rye chromatin in wheat background during the individual steps of breeding programs, C-banding, *in situ* hybridization, and the use of PCR-based markers have been exploited with great success. However, the application of C-banding technology in breeding programs is limited because of its time consuming nature and a required high skill level in interpreting results^7^. PCR-based markers and *in situ* hybridization have been widely used to detect rye chromatin in wheat-rye hybrids. The PCR-based markers are convenient for identifying rye chromatin, however, they do not show the physical size of the rye translocations. Fluorescence *in situ* hybridization (FISH) and genomic *in situ* hybridization (GISH) have been most useful for investigating wheat-rye hybrids^8^,^9^,^10^,^11^. FISH can be used to investigate different morphologies and the organization of chromosomes^12^. GISH has the added advantage of determining the size and breakpoint positions of wheat-rye translocations as well as providing a direct visual method for distinguishing parental genomes and analyzing genome organization in these hybrids. Genomic DNA of rye is routinely used in GISH as a probe but the labeling of the DNA is time-consuming and labor-intensive. Recently, synthetic oligonucleotides carrying a fluorescent label were developed for FISH analysis of wheat and rye chromosomes^8^,^9^,^13^,^14^, making the process easier and more economical^14^. This has led to the development of an even simpler and more efficient technique termed non-denaturing FISH (ND-FISH) analysis^15^,^16^. However, despite probes such as Oligo-pSc119.2-1, Oligo-pSc119.2-2, (GACA)n and (AAC)n being able to detect parts of all rye chromosomes^8^,^9^,^14^, GISH is still required to elucidate the size and positioning of rye introgressions. In order to overcome some obstacles connected with GISH assays using labeled genomic DNAs as probe, we report here the development and application of three oligonucleotide probes that can be used for ND-FISH assays and eliminate the requirement of GISH to discriminate rye from wheat chromosomes. In addition, some oligonucleotide probes that were developed previously^14^ have also been tested for ND-FISH analysis.

## Results

**Figure 1:**
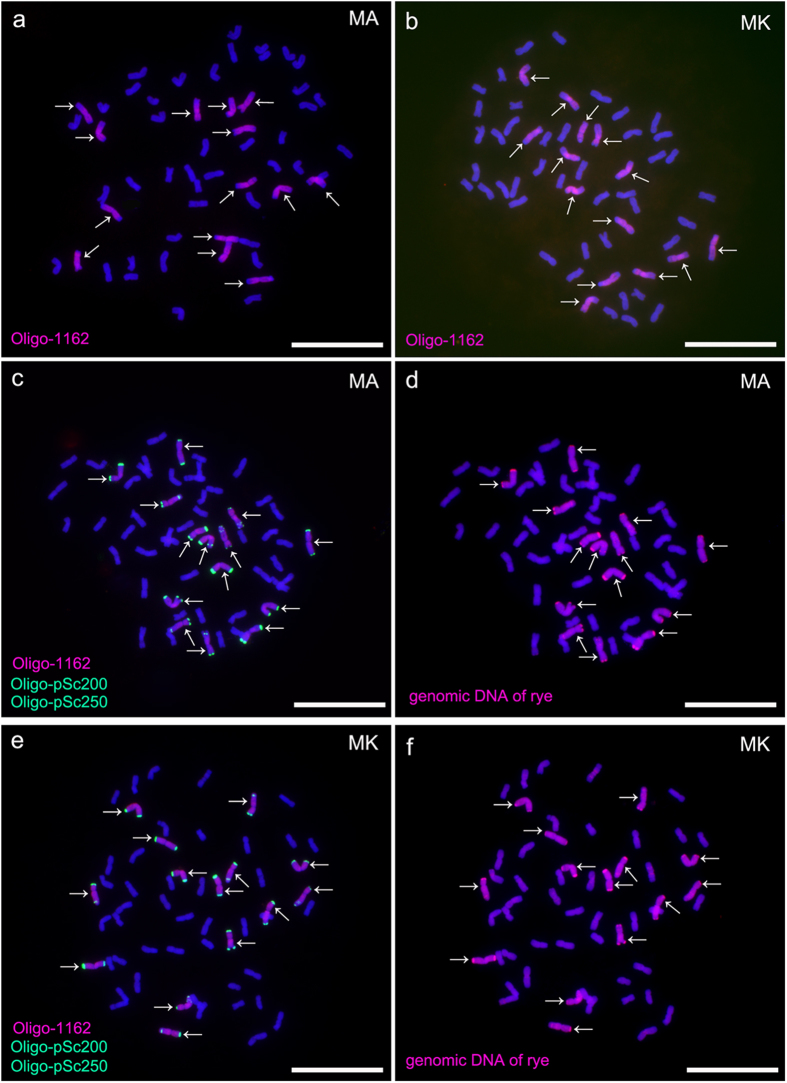
ND-FISH experiments on triticale chromosomes performing with Oligo-1162 as probe (red) showing the 14 rye-origin chromosomes of octoploid triticale MA (**a**) and octoploid triticale MK (**b**). Sequential ND-FISH and GISH experiments on octoploid triticale chromosomes performing with Oligo-1162 (red), Oligo-pSc200 (green), Oligo-pSc250 (green) and genomic DNA of rye (red) as probes showing the 14 rye-origin chromosomes of MA (**c,d**) and MK (**e,f**). Arrows indicate the rye-origin chromosomes. Chromosomes were counterstained with DAPI (blue). *Scale bar* 10 μm.

Octoploid triticales derived from the crosses *T. aestivum* L. Mianyang11 × *S. cereale* L. AR106BONE (MA) and *T. aestivum* L. Mianyang11 ×* S. cereale* L. Kustro (MK) were analyzed by ND-FISH using oligonucleotides Oligo-1162, Oligo-pSc200 and Oligo-pSc250 as probes (Table 1). Oligo-1162 produced hybridization signals on all 14 rye chromosomes in both octoploid triticales MA and MK, but no strong signals were observed on the 42 wheat chromosomes (Fig. 1a,b). This result indicated that the use of Oligo-1162 allows rye chromosomes to be unambiguously distinguished from wheat chromosomes. However, strong signals from Oligo-1162 were not produced on heterochromatic blocks (Fig. 1a,b). The combining of Oligo-1162, Oligo-pSc200 and Oligo-pSc250 probes together painted almost the entire rye genome including the telomeric and sub-telomeric heterochromatic blocks (Fig. 1c,e). The signals of Oligo-pSc200 and Oligo-pSc250 can not be observed on wheat chromosomes (Fig. 1c,e). Subsequent GISH analysis on the same cells in Fig. 1c and Fig. 1e indicated that the signal patterns of genomic DNA of rye were similar to the combined patterns of Oligo-1162, Oligo-pSc200 and Oligo-pSc250 (Fig. 1c–f). The heterochromatic blocks reflected by rye genomic DNA corresponded to the same places where Oligo-pSc200 and Oligo-pSc250 produced signals (Fig. 1c–f). These results showed that Oligo-1162, Oligo-pSc200 and Oligo-pSc250 can be used together for ND-FISH assays to replace genomic DNA of rye as probes to distinguish rye from wheat chromosomes.

**Figure 2:**
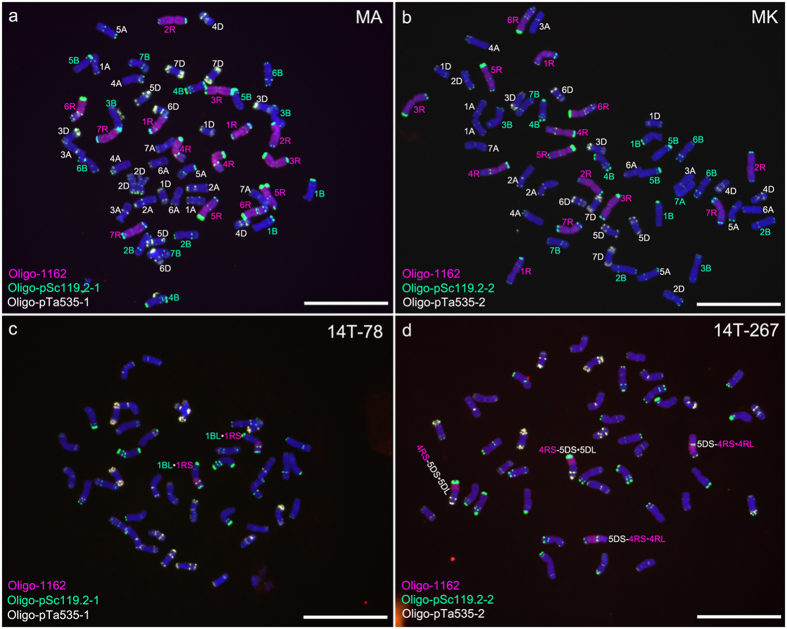
ND-FISH experiments on chromosomes of octoploid triticales and wheat-rye translocation lines performing with Oligo-1162 (red), Oligo-pSc119.2-1 (green), Oligo-pSc119.2-2 (green), Oligo-pTa535-1 (white) and Oligo-pTa535-2 (white) as probes. **a** Signals of Oligo-1162, Oligo-pSc119.2-1 and Oligo-pTa535-1 on chromosomes of octoploid triticale MA. **b** Signals of Oligo-1162, Oligo-pSc119.2-2 and Oligo-pTa535-2 on chromosomes of octoploid triticale MK. **c** Signals of Oligo-1162, Oligo-pSc119.2-1 and Oligo-pTa535-1 on chromosomes of wheat-rye 1BL·1RS translocation line 14T-78. **d** Signals of Oligo-1162, Oligo-pSc119.2-2 and Oligo-pTa535-2 on chromosomes of wheat-rye 5DS-4RS·4RL and 4RS-5DS·5DL translocation line 14T-267. Chromosomes were counterstained with DAPI (blue). *Scale bar* 10 μm.

**Figure 3:**
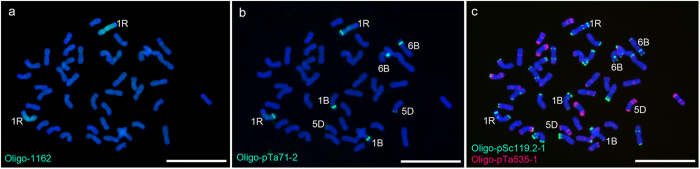
Sequential ND-FISH experiments on chromosomes of wheat-rye 1R disomic addition line 14T-71 performing with Oligo-1162 (green), Oligo-pTa71-2 (green), Oligo-pSc119.2-1 (green) and Oligo-pTa535-1 (red) as probes. **a** Oligo-1162 signals on chromosomes of line 14T-71. **b** Oligo-pTa71-2 signals on the same cell in (**a**). **c** Oligo-pSc119.2-1 and Oligo-pTa535-1 signals on the same cell in (**a**) and (**b**). Chromosomes were counterstained with DAPI (blue). *Scale bar* 10 μm.

**Figure 4:**
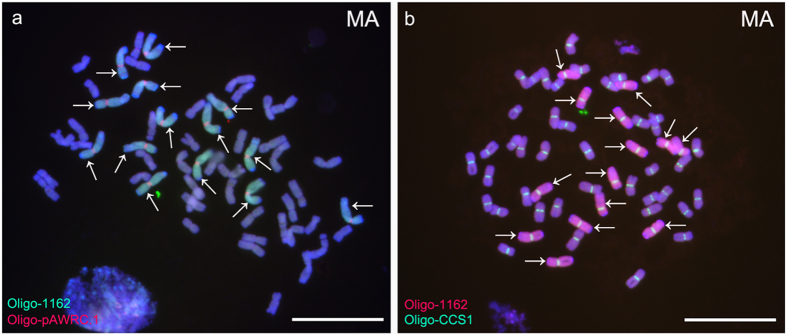
ND-FISH experiments on chromosomes of octoploid triticale MA performing with Oligo-1162 (green), Oligo-pAWRC.1 (red) and Oligo-CCS1 (green) as probes. **a** Oligo-1162 and Oligo-pAWRC.1 signals on chromosomes of octoploid triticale MA. **b** Oligo-1162 and Oligo-CCS1 signals on chromosomes of octoploid triticale MA. Arrows indicate the rye-origin chromosomes. Chromosomes were counterstained with DAPI (blue). *Scale bar* 10 μm.

Additionally, Oligo-pSc119.2-1, Oligo-pSc119.2-2, Oligo-pTa535-1, Oligo-pTa535-2, Oligo-pTa71-2, Oligo-pAWRC.1 and Oligo-CCS1^14^ were tested with ND-FISH assays. Oligo-1162 was combined with Oligo-pSc119.2-1 and Oligo-pTa535-1 distinguished all wheat and rye chromosomes in the octoploid triticale MA (Fig. 2a). Substituting Oligo-pSc119.2-1 and Oligo-pTa535-1 for Oligo-pSc119.2-2 and Oligo-pTa535-2, whilst maintaining Oligo-1162, facilitated the identification of all wheat and rye chromosomes in MK (Fig. 2b). The signal patterns of Oligo-pSc119.2-1, Oligo-pSc119.2-2, Oligo-pTa535-1 and Oligo-pTa535-2 on wheat or rye chromosomes were in agreement with Tang *et al.*^14^. Combined with Oligo-1162, Oligo-pSc119.2-1, Oligo-pSc119.2-2, Oligo-pTa535-1 and Oligo-pTa535-2 can also identify wheat-rye 1BL·1RS translocation chromosomes in the wheat line 14T-78 (Fig. 2c) and the 5DS-4RS·4RL and 4RS-5DS·5DL translocations in the wheat line 14T-267 (Fig. 2d). In addition, by using ND-FISH analysis, Oligo-pTa71-2 hybridized well to 1B, 6B, 5D and 1R chromosomes in the wheat-rye disomic addition line 14T-71(Fig. 3), Oligo-pAWRC.1 only produced strong signals on centromeric regions of rye chromosomes and Oligo-CCS1 hybridized to the centromeric regions of both rye and wheat chromosomes (Fig. 4).

## Discussion

Rye is a useful genetic resource in wheat breeding as rye translocations can be incorporated into wheat relatively easily, bringing with it many important genes. However, it is important to be able to differentiate and localize rye genetic material in the wheat background. The GISH technique using rye genomic DNA as a probe has been widely used to do this^5^,^11^,^17^. However, in the routine procedure of GISH, genomic DNA of rye had to be extracted from leaves of rye plants and labeled by nick translation. In addition, both chromosomes and probes need to be denatured before hybridization and hybridization typically occurs several hours. Thus the routine procedure of GISH using genomic DNA of rye as a probe is time-consuming and labor-intensive. In the present study, the synthetic oligonucleotide probe Oligo-1162 was used to identify rye chromosomes in wheat backgrounds. However, it did not produce strong signals on heterochromatic regions of rye, possibly because Oligo-1162 is not a tandem repeated sequence. Such repeated sequences are common in heterochromatic regions of rye chromosomes. Conversely the probes Oligo-pSc200 and Oligo-pSc250 did produce signals in these heterochromatic regions and by combining with Oligo-1162, this ND-FISH assays can replace genomic DNA of rye. This method is convenient as designed oligonucleotides can be commercially labeled with fluorochrome, eliminating the nick-translation procedure. Furthermore, synthesized oligonucleotide probes are much cheaper than genomic DNA probes of rye (Table 2), and the use of ND-FISH analysis with oligo-probes reduces the hybridization time to one hour. In addition, ND-FISH does not require the denaturation of chromosomes, which can avoid potential structural alterations of the chromosome morphology. The use of these three oligonucleotide probes greatly simplifies the detection of rye chromosomes in wheat backgrounds.

Previous work by Tang *et al.*^14^ resulted in the development of the probes, Oligo-pSc119.2-1, Oligo-pSc119.2-2, Oligo-pTa535-1, Oligo-pTa535-2, Oligo-pTa71-2, Oligo-pAWRC.1 and Oligo-CCS1. These oligonucleotide probes proved that they can replace the repetitive sequences pSc119.2, pTa-535, pTa71, pAWRC.1, and CCS1 as probes for FISH analysis of wheat and rye^14^. In the present study, these oligonucleotide probes proved that they were suitable for ND-FISH analysis and the hybridization time was also reduced to one hour. Therefore, it is now more convenient to use the oligonucleotide probes to analyze chromosomes of wheat and rye. ND-FISH has already been used to analyze chromosomes of plants and *Drosophila melanogaster*^15^,^16^,^18^,^19^. In these previous studies, simple sequence repeats (SSRs) were used as oligonucleotide probes for ND-FISH analysis^15^,^16^,^18^,^19^. In the present study, the oligonucleotide probes were not SSRs. The results in this study indicate that the non-SSR oligonucleotides also have the ability to invade chromosomes. The precise mechanisms about the chromosome invasion by oligonucleotides are unclear though Cuadrado and Jouve have already discussed this issue in detail^16^.

In conclusion, the oligonucleotide probes Oligo-1162, Oligo-pSc200 and Oligo-pSc250 can be used for ND-FISH assays to detect rye chromosomes in wheat backgrounds and wheat-rye translocation chromosomes. Oligonucleotide probes Oligo-pSc119.2-1, Oligo-pSc119.2-2, Oligo-pTa535-1, Oligo-pTa535-2, Oligo-pTa71-2, Oligo-pAWRC.1 and Oligo-CCS1 can also be used for ND-FISH of wheat and rye. By using ND-FISH, the hybridization time of these oligonucleotide probes was reduced to one hour. The oligonucleotide probes have provided an easier, faster and more cost-effective method for the studying of wheat and hybrids derived from wheat × rye.

## Methods

### Plant materials

The plant materials used in this study included octoploid triticales MA and MK, derived from *T. aestivum* L. Mianyang11 (genomes AABBDD) × *S. cereale* L. AR106BONE (genome RR) and Mianyang11 × *S. cereale* L. Kustro (genome RR), respectively. The wheat-rye 1R disomic addition line 14T-71, the wheat-rye 1BL·1RS translocation line 14T-78 and the wheat-rye 5DS-4RS·4RL and 4RS-5DS·5DL translocation line 14T-267 were also used. Lines 14T-71 and 14T-78 were slected from the progeny that were derived form MK × Mianyang11. Some MK seeds were irradiated with fast neutron. Then the irradiated MK was used as recipient to cross with common wheat cultivar CN27 (genomes AABBDD) and line 14T-267 was selected form the progeny of this cross combination. The detailed information of these materials are listed in Table 3.

### Obtaining new oligonucleotide probes detecting rye chromosomes

Genomic DNA of *S. cereale* L. Kustro was sequenced by using the Specific Length Amplified Fragment Sequencing (SLAF-seq) method (Biomarker, Beijing, China). The sequencing procedure was performed as described by Chen *et al.*^20^ with some modifications. Briefly, genomic DNA of Kustro was digested with restriction enzyme HaeIII, and the reads were compared with the sequences of wheat A genome, D genome and *T. aestivum* L. Chinese Spring. Then, a lot of reads, which were 60 bp long and had low wheat homology were obtained. One read with a sequence depth of 85 was randomly selected to use as an oligonucleotide probe. This probe was named Oligo-1162 (Table 1). In addition, two oligonucleotide probes Oligo-pSc200 and Oligo-pSc250 (Table 1) were developed according to the tandem repetitive DNA sequences pSc200 and pSc250^21^, respectively. The names and the sequences of these probes are listed in Table 1.

### ND-FISH analysis

The synthetic oligonucleotide probes including Oligo-1162, Oligo-pSc200, Oligo-pSc250, Oligo-pSc119.2-1, Oligo-pSc119.2-2, Oligo-pTa535-1, Oligo-pTa535-2, Oligo-pTa71-2, Oligo-pAWRC.1 and Oligo-CCS1 were used for ND-FISH analysis. The oligonucleotide probes were 5`end-labelled with 6-carboxyfluorescein (6-FAM), 6-carboxytetramethylrhodamine (Tamra) or Cy5. The signals of probes labeled with 6-FAM, Tamra and Cy5 were displayed in the colors green, red and white, respectively. Oligonucleotide probes were synthesized by Shanghai Invitrogen Biotechnology Co. Ltd. (Shanghai, China). These synthesized probes were diluted by using 1×TE solution (pH7.0). For Oligo-1162, Oligo-pSc200 and Oligo-pSc250, probe amounts per slide are listed in Table 1. For Oligo-pSc119.2-1, Oligo-pSc119.2-2, Oligo-pTa535-1, Oligo-pTa535-2, Oligo-pTa71-2, Oligo-pAWRC.1 and Oligo-CCS1, probe amounts per slide were as described by Tang *et al.*^14^. The chromosome spreads of materials were prepared with the methods described by Han *et al.*^22^. The probe mixture (each probe in 2 × SSC and 1 × TE buffer, pH7.0, total volume = 10 μl) was dropped at the center of the cell spreads and covered with glass coverslip. Slides were stored in a moist box at 42 °C for 1 h and washed in 2 × SSC at room temperature. The slides were mounted with Vectashield mounting medium (Vector Laboratories) with DAPI (4`,6-diamidino-2-phenylindole).

### Sequential *in situ* hybridization

Probes Oligo-1162, Oligo-pSc200 and Oligo-pSc250 were hybridized together to mitotic metaphase chromosomes of octoploid triticales MA and MK in the ND-FISH method. After rinsing the chromosome preparations of MA and MK, GISH was carried out on the same slides with the genomic DNA probe of Kustro. Genomic DNA of rye was labeled with Texas Red-5-dUTP (Invitrogen). Probe labeling and *in situ* hybridization were conducted with the methods described by Han *et al.*^22^. In addition, sequential ND-FISH was used to analyze wheat-rye 1R disomic addition line 14T-71. Probe Oligo-1162 was hybridized to mitotic metaphase chromosomes of 14T-71, probe Oligo-pTa71-2 was hybridized to the same slide after rising the chromosome preparations. Finally, probes Oligo-pSc119.2-1 and Oligo-pTa535-1 were hybridized to the same slide after the second rising of the chromosome preparations.

Images were taken using an epifluorescence microscope (BX51, Olympus) equipped with a cooled charge-coupled device camera operated with HCIMAGE Live software (version 2.0.1.5) and processed with photoshop CS 3.0.

## Author Contributions

S.F. and Z.T. designed the study, analyzed the data and wrote the manuscript. L.C., Y.W., M.L., L.Q. and B.Y. performed the experiments. Z.Y. and Z.R. discussed the manuscript.

## Additional Information

**How to cite this article**: Fu, S. *et al.* Oligonucleotide Probes for ND-FISH Analysis to Identify Rye and Wheat Chromosomes. *Sci. Rep.* **5**, 10552; doi: 10.1038/srep10552 (2015).

## Figures and Tables

**Table 1:** Sequences of synthesized oligonucleotide probes for detecting rye chromosomes.

**Probe names**	**Nucleotide sequences of probes (5`-3`)**	**Amount applied to ND-FISH analysis (ng/slide)**
Oligo-1162	TGTGGCTTTA TGTTGTTTTG GTATCTTTCT TTTGGATCTT CACCCGTAGTCGGGTTGT	11.93
Oligo-pSc200	CTCACTTGCT TTGAGAGTCT CGATCAATTC GGACTCTAGG TTGATTTTTG TATTTTCT	0.584
Oligo-pSc250	TGTGTTGTTC TTGGACAAAA CAATGCATAC CATCTCTTCT AC	11.28

**Table 2:** Comparison of costing between preparing oligonucleotides and genomic DNA of rye as probes.

**Name of probe**	**Amount of probe (μg)**	**Number of slides that can be detected (piece)**	**Costing of probe ($/slide)**^a^	**Total costing of probe for per slide ($/slide)**^a^
Oligo-1162	149.10	12500.00	0.0056-0.0205	0.0113-0.0417		
Oligo-pSc200	146.10	250000.00	0.0003-0.0010			
Oligo-pSc250	140.95	12500.00	0.0054-0.0202			
Rye genomic DNA	100.00	5000.00	0.50-0.60	0.50-0.60		

^a^The costing is valued at present market prices in China and the costing changes according to the sequence length of probe and the labeled fluorochrome.

**Table 3:** Germplasm used in this study.

**Name**	**Genetic structure**	**Pedigree**	**Chromosome composition**	**Chromosome number**
MA	octoploid triticale	Mianyang11(AABBDD) × AR106BONE(RR)	42 wheat chromosomes, 14 rye chromosomes	56
MK	octoploid triticale	Mianyang11(AABBDD) × Kustro(RR)	42 wheat chromosomes, 14 rye chromosomes	56
14T-71	wheat-rye 1R disomic addition line	MK × Mianyang11	42 wheat chromosomes, two rye 1R chromosomes	44
14T-78	wheat-rye 1BL·1RS translocation line	MK × Mianyang11	40 wheat chromosomes, two 1BL·1RS translocation chromosomes	42
14T-267	wheat-rye 5DS-4RS·4RL and 4RS-5DS·5DL translocation line	Irradiated MK × CN27 (a common wheat cultivar, AABBDD)	40 wheat chromosomes, two 5DS-4RS·4RL and two 4RS-5DS·5DL translocation chromosomes	44

## References

[b1] Friebe B., Jiang J., Raupp W. J., McIntosh R. A. & Gill B. S. Characterization of wheat-alien translocation conferring resistance to diseases and pests: current status. Euphytica 91, 59–87 (1996).

[b2] Lukaszewski A. J., Porter D. R., Baker C. A., Rybka K. & Lapinski B. Attempts to transfer Russian wheat aphid resistance from a rye chromosome in Russian triticales to wheat. Crop Sci. 41, 1743–1749 (2001).

[b3] Kim W., Johnson J. W., Baenziger P. S., Lukaszewski A. J. & Gaines C. S. Agronomic effect of wheat-rye translocation carrying rye chromatin (1R) from different sources. Crop Sci. 44, 1254–1258 (2004).

[b4] Camacho M. V., Matos M., González C., Pérez-Flores V. & Pernaute B. *Secale cereale* inter-microsatellites (SCIMs): chromosomal location and genetic inheritance. Genetica 123, 303–311 (2005). 15954501 10.1007/s10709-004-5553-z

[b5] An D. G. *et al.* Molecular cytogenetic characterization of a new wheat-rye 4R chromosome translocation line resistant to powdery mildew. Chromosome Res. 21, 419–432 (2013). 23836161 10.1007/s10577-013-9366-8

[b6] Rabinovich S. V. Importance of wheat-rye translocations for breeding modern cultivars of *Triticum aestivum* L. Euphytica 100, 323–340 (1998).

[b7] Rayburn A. L. & Gill B. S. Molecular identification of the D-genome chromosomes of wheat. J. Hered. 77, 253–255 (1986).

[b8] Cuadrado Á. & Schwarzacher T. The chromosomal organization of simple sequence repeats in wheat and rye genomes. Chromosoma 107, 587–594 (1998). 9933412 10.1007/s004120050345

[b9] Cuadrado Á. & Jouve N. Evolutionary trends of different repetitive DNA sequences during speciation in the genus *Secale*. J. Hered. 93, 339–345 (2002). 12547922 10.1093/jhered/93.5.339

[b10] Contento A., Heslop-Harrison J. S. & Schwarzacher T. Diversity of a major repetitive DNA sequence in diploid and polyploid Triticeae. Cytogenet. Genome Res. 109, 34–42 (2005). 15753556 10.1159/000082379

[b11] Fu S. L., Yang M. Y., Ren Z. L., Yan B. J. & Tang Z. X. Abnormal mitosis induced by wheat-rye 1R monosomic addition lines. Genome 57, 21–28 (2014). 24564212 10.1139/gen-2013-0115

[b12] Heckmann S. *et al.* The holocentric species Luzula elegans shows interplay between centromere and large-scale genome organization. Plant J. 73, 555–565 (2013). 23078243 10.1111/tpj.12054

[b13] Danilova T. V., Friebe B. & Gill B. S. Single-copy gene fluorescence *in situ* hybridization and genome analysis: Acc-2 loci mark evolutionary chromosomal rearrangements in wheat. Chromosoma 121, 597–611 (2012). 23052335 10.1007/s00412-012-0384-7

[b14] Tang Z. X., Yang Z. J. & Fu S. L. Oligonucleotides replacing the roles of repetitive sequences pAs1, pSc119.2, pTa-535, pTa71, CCS1, and pAWRC.1 for FISH analysis. J. Appl. Genet. 55, 313–318 (2014). 24782110 10.1007/s13353-014-0215-z

[b15] Cuadrado A., Golczyk H. & Jouve N. A novel, simple and rapid nondenaturing FISH (ND-FISH) technique for the detection of plant telomeres. Potential used and possible target structures detected. Chromosome Res. 17, 755–762 (2009). 19669910 10.1007/s10577-009-9060-z

[b16] Cuadrado Á. & Jouve N. Chromosomal detection of simple sequence repeats (SSRs) using nondenaturing FISH (ND-FISH). Chromosoma 119, 495–503 (2010). 10.1007/s00412-010-0273-x 20393739

[b17] Ribeiro-Carvalho C., Guedes-Pinto H., Heslop-Harrison J. S. & Schwarzacher T. Introgression of rye chromatin on chromosome 2D in the Portuguese wheat landrace ‘Barbela’. Genome 44, 1122–1128 (2001). 11768216

[b18] Cuadrado Á. & Jouve N. Novel simple sequence repeats (SSRs) detected by ND-FISH in heterochromatin of *Drosophila melanogaster*. BMC Genomics 12, 10.1186/1471-2164-12-205 (2011). PMC3114746 21521504

[b19] Pavia I., Carvalho A., Rocha L., Gaspar M. J. & Lima-Brito J. Physical location of SSR regions and cytogenetic instabilities in *Pinus sylvestris* chromosomes revealed by ND-FISH. J. Genet. 93, 567–571 (2014). 25189261 10.1007/s12041-014-0412-x

[b20] Chen S. Q. *et al.* The development of 7E chromosome-specific molecular markers for *Thinopyrum elongatum* based on SLAF-seq technology. PLoS ONE 8, e65122. 10.1371/journal.pone.0065122 (2013). 23762296 PMC3677899

[b21] Vershinin A. V., Schwarzacher T. & Heslop-Harrison J. S. The large-scale genomic organization of repetitive DNA families at the telomeres of rye chromosomes. Plant Cell 7, 1823–1833 (1995). 8535136 10.1105/tpc.7.11.1823 PMC161041

[b22] Han F. P., Lamb J. C. & Birchler J. A. High frequency of centromere inactivation resulting in stable dicentric chromosomes of maize. Pro. Natl. Acad. Sci. USA. 103, 3238–3243 (2006). 10.1073/pnas.0509650103 PMC1413895 16492777

